# Infections Combined With Myelin Oligodendrocyte Glycoprotein Antibody-Associated Disease: A Case Report and Systematic Review of the Literature

**DOI:** 10.7759/cureus.71229

**Published:** 2024-10-10

**Authors:** Huiyao Xiang, Moushan Cai

**Affiliations:** 1 Department of Neurology, The First College of Clinical Medical Science, China Three Gorges University, Yichang, CHN

**Keywords:** central nervous system, clinical features, immunoglobulin, infections, myelin-oligodendrocyte glycoprotein

## Abstract

Myelinating oligodendrocyte glycoprotein antibody-associated disease (MOGAD) is an immune-mediated inflammatory demyelinating disease of the central nervous system. Its specific etiology and pathogenesis remain unclear. In recent years, there have been increasing reports of MOGAD occurring after infections. Even cases of concurrent infection and MOGAD have been documented. We report a clinical case of a 14-year-old male patient admitted to the hospital with a fever and loss of consciousness. He underwent thorough medical examinations. The results of second-generation sequencing of the metagenome of the cerebrospinal fluid revealed that he was infected with Haemophilus parainfluenzae, and serum testing showed positive MOG antibodies. He was discharged after improving with intravenous immunoglobulin and other treatments.

## Introduction

Myelin oligodendrocyte glycoprotein (MOG) is a protein found only in the outer layers of myelin and oligodendrocyte membranes in the central nervous system (CNS). Myelin oligodendrocyte glycoprotein antibody-associated disease (MOGAD) is a recently identified autoimmune disorder that can cause CNS demyelination in both adults and children. It is considered separate from multiple sclerosis and optic neuromyelitis optica spectrum disorders [1]. MOGAD can be preceded by a predisposing factor, such as infection, in 37%-70% of cases, with viral infections being the most common [2]. We report a case of Haemophilus parainfluenzae infection combined with MOGAD, summarize the relevant literature, research the clinical features, and improve the understanding of this disease for early diagnosis and treatment.

## Case presentation

The patient is a 14-year-old male who visited the clinic on October 26, 2023, complaining of fever for five days and loss of consciousness on one occasion. Since the onset of the illness, his temperature had reached a maximum of 38.9℃, accompanied by a sore throat and cough. He experienced a brief loss of consciousness at the time of presentation with symptoms that included double gaze and limb twitching, which lasted approximately one minute. There were no signs of foaming at the mouth or incontinence. We consider that he experienced a seizure. Neurologic examination revealed nuchal rigidity and positive Koenig's sign, and the remainder of the patient's physical exam was unremarkable.

Laboratory examination showed negative *Mycobacterium tuberculosis* DNA and T-cell tests, respiratory pathogens tests, respiratory virus nucleic acid tests, blood cultures, thyroid function, tumor markers, autoantibody profile, antibodies to human immunodeficiency virus antigens, *Syphilis* antibodies, and antibodies to immunoglobulins G4. A computed tomography scan of the entire abdomen, electroencephalogram, magnetic resonance imaging (MRI) of the brain scanning and enhancement, optic nerves, and whole spinal cord revealed no abnormalities. The cerebrospinal fluid (CSF) examination revealed a pressure of 210 mmH_2_O, a leukocyte count of 522×10^6^/L, and a protein level of 101.50 mg/dl. Tests such as glucose and chloride levels in CSF, antacid staining, ink staining, cryptococcal capsular antigen detection, and CSF culture were also normal. However, the results of second-generation sequencing of the metagenome of the CSF suggested the presence of *Haemophilus parainfluenzae* infection. The serum and CSF tested positive for MOG antibodies (titers of 1:640 and 1:320, by the cell-based assay method). In addition, other tests, such as those for autoimmune encephalitis antibodies, paraneoplastic syndrome-related antibodies, AQP4 antibodies, and oligoclonal bands in the CSF and blood, were normal (detailed information is given in Tables 1, 2).

**Table 1:** Blood-related examination items and results

Test items	Test results	Unit	Normal range
Mycobacterium tuberculosis DNA tests	Negative		Negative
Mycobacterium tuberculosis T-cell tests	Negative		Negative
Respiratory virus nucleic acid tests（Adenovirus, influenza A virus, influenza B virus, respiratory syncytial virus, parainfluenza virus I, parainfluenza virus III）	Negative		Negative
Respiratory pathogens tests（Adenovirus, respiratory syncytial virus, influenza virus A, influenza virus B, parainfluenza virus, Mycoplasma pneumoniae, Chlamydia pneumoniae and Legionella pneumophila）	Negative		Negative
Blood cultures	Negative		Negative
Hypersensitive thyroglobulin	26.72	ng/ml	3.5-77
Thyroid peroxidase antibodies	3.83	IU/ml	0-30
Thyrotropin receptor antibodies	0.25	IU/L	＜1.5
Thyroglobulin antibodies	9.62	%	0-30
Thyroid microsomal antibodies	8.90	%	0-20
Triiodothyronine	1.10	nmol/L	0.89-2.45
Thyroxine	79.92	nmol/L	62.68-150.84
Thyroid-stimulating hormone	3.23	uIU/ml	0.35-4.94
Free triiodothyronine	3.50	pmol/L	2.43-6.01
Free thyroxine	11.77	pmol/L	9.01-19.05
Carbohydrate antigen 50	＜1.00	U/ml	＜21.96
Alpha-fetoprotein	3.12	ng/ml	＜7.42
Carcinoembryonic antigen	2.80	ng/ml	＜4.1
Carbohydrate antigen 125	29.81	U/ml	＜37.22
Carbohydrate antigen 153	17.75	U/ml	＜27.96
Carbohydrate antigen 199	＜2.60	U/ml	＜27.50
Carbohydrate antigen 242	＜0.50	U/ml	＜10.00
Neuron specific enolase	8.25ng/ml	ng/ml	＜16.01
Autoantibodies 19 items	Negative		Negative
Antibodies to human immunodeficiency virus antigens	0.01 S/CO		0-0.99
Syphilis antibodies	0.01 S/CO		0-0.99
Antibodies to immunoglobulins G4	960.37	mg/L	110.0-1570.0
Myelin oligodendrocyte glycoprotein antibodies	1:640		Negative
Autoantibodies to aquaporin 4 antibodies	Negative		Negative
Oligoclonal bands	Negative		Negative
Autoimmune encephalitis antibodies 21 items	Negative		Negative
Paraneoplastic syndrome-related antibodies (anti⁃neuronal nuclear antibody type⁃1, anti⁃neuronal nuclear antibody type⁃2, collapsin response⁃mediator protein⁃5, anti-neuronal nuclear antibodies-3, purkinje cell cytoplasmic antibody type 2 antibodies)	Negative		Negative

**Table 2:** CSF-related examination items and results

Test items	Test results	Unit	Normal range
Leukocyte count	522	10^6^/L	0-8
Protein	101.50	mg/dl	15-45
Glucose	2.38	mmol/L	2.2-3.9
Chloride	126.3	mmol/L	118-132
Antacid staining	Negative		Negative
Ink staining	Negative		Negative
Cryptococcal capsular antigen	Negative		Negative
Cultures	Negative		Negative
Second-generation sequencing of the metagenome	Hemophilus parainfluenzae		Negative
Myelin oligodendrocyte glycoprotein antibodies	1:320		Negative
Autoantibodies to aquaporin 4 antibodies	Negative		Negative
Oligoclonal bands	Negative		Negative
Autoimmune encephalitis antibodies 21 items	Negative		Negative
Paraneoplastic syndrome-related antibodies (anti⁃neuronal nuclear antibody type⁃1, anti⁃neuronal nuclear antibody type⁃2, collapsin response⁃mediator protein⁃5, anti-neuronal nuclear antibodies-3, purkinje cell cytoplasmic antibody type 2 antibodies)	Negative		Negative

The patient received immunoglobulin treatment at a daily dose of 0.4 g/(kg-d) for five days, followed by 20 mg prednisone in the morning. During hospitalization, the patient received additional treatments including infection control with cefotaxime 2.0g every 12 hours, intracranial pressure reduction, gastroprotection, potassium and calcium supplementation, and nutritional support. After four weeks, the patient showed no positive neurological signs, and a repeat serum anti-MOG antibody test was performed at a ratio of 1:320, improved from pre-admission. After discharge, the patient was followed up regularly, with the last follow-up scheduled for the end of June 2024, and no recurrence of the disease was observed, while the serum anti-MOG antibody test was negative.

## Discussion

MOG protein is expressed in the outermost layer of myelin and oligodendrocyte membranes in the CNS. It is a major protein component of myelin, that is highly immunogenic and was first detected by immunoblotting and enzyme-linked immunosorbent assay 30 years ago [3,4]. The exact function of MOG is not yet fully understood, but its presence in mature oligodendrocytes suggests that it plays a role in their maturation and in maintaining the structural integrity of myelin and cell surface interactions [5]. Anti-MOG autoantibodies (MOG-IgG) are predominantly of the IgG1 subtype and are categorized as pathogenic or nonpathogenic [2].

Recently, there has been a gradual increase in the number of case reports of preexisting coinfections in MOG antibody-positive patients, especially those caused by viral pathogens [6,7]. In this case, the patient initially experienced fever and classical features of an encephalopathy syndrome. CSF examination revealed increased leukocyte counts and protein levels. CSF second-generation metagenome sequencing revealed infection with *Haemophilus parainfluenzae*, while the serum tested strongly positive for MOG-IgG. This ultimately led to the diagnosis of *Haemophilus parainfluenzae* infection combined with MOGAD.

The presence of serum MOG-IgG is crucial for diagnosing MOGAD and is highly specific [8]. The titer of MOG-IgG correlated with the positive predictive value, specificity, disease severity, and relapse rate. Patients with high or consistently positive serum MOG-IgG titers are more likely to experience relapses. However, caution is needed when diagnosing MOGAD if the antibody level is less than 1:20 and if the clinical symptoms are atypical [8,9]. In this case, the patient had a MOG-IgG titer of 1:640 and had typical clinical manifestations of encephalopathy syndrome. Other suspected diseases were excluded, so the diagnosis could be made more reliably.

To explore the relationship between infection and MOGAD in-depth, we conducted a comprehensive search of several databases, including the China National Knowledge Infrastructure (CNKI), Wanfang Database, Web of Science, and PubMed, to collect existing relevant literature. Ultimately, we included 55 studies related to infections combined with MOGAD, covering the clinical data of 65 patients. Among them, 39 were males and 27 were females, resulting in a male-to-female ratio of 1.44:1. The mean age of disease onset was 34.77 years. After systematically analyzing the literature, we discovered that there were various sources of infection [7]. Viral infections, particularly coronavirus disease 2019 (COVID-19), had a significant impact. In our study, there were 33 cases of COVID-19 infection, accounting for 50% of the total. Additionally, five cases (7.57%) were infected with COVID-19 vaccination; four cases (6.06%) were infected with Mycoplasma pneumoniae. There were three cases each of human immunodeficiency virus (HIV) infection and varicella-zoster virus infection (4.55%). Two cases involved other viruses such as cytomegalovirus, influenza A, and Epstein-Barr (3.03%). Furthermore, there were rare cases of coinfections, including COVID-19 and human herpesvirus type 6, Zika virus, mumps virus, genital herpes simplex virus, adenovirus, rubella virus, Diphtheria-tetanus-pertussis vaccine, dengue virus, Treponema spirochetes, Chlamydia pneumonia, Brucella melitensis, and Haemophilus parainfluenzae, each occurring in one case (1.52%). The most common clinical manifestations are optic neuritis and myelitis [10]. In our study, the main clinical manifestations observed were as follows: optic neuritis in 19 cases (28.79%), acute disseminated encephalomyelitis in 15 cases (22.73%), myelitis in 15 cases (22.73%), neuromyelitis optica in eight cases (12.12%), encephalitis in seven cases (10.61%), and two cases of meningoencephalitis (3.03%). Notably, one patient with Chlamydia pneumoniae infection presented with disseminated encephalomyelitis with a multiphasic course, whereas another patient with novel coronavirus infection presented with optic neuritis combined with Guillain-Barré syndrome. Among the main clinical symptoms, the most common presentations included fever (50.00%), visual disturbances (43.94%), urinary retention (40.91%), sensory disturbances (36.36%), paraplegia (31.82%), headache (25.76%), eye pain (22.73%), ataxia (15.15%), and limb weakness (12.12%). Additionally, several less prevalent symptoms, each occurring in less than 10% of cases, including hallucinations, dysarthria, and dysphagia, were detected. Imaging lacks specificity, and the brain, optic nerves, and whole spinal cord can be affected [10,11]. In our study, 62 patients underwent at least one MRI examination. The results revealed that 38 cases (61.29%) had spinal cord lesions, with the highest incidence in the thoracic medulla (68.42%). Additionally, 30 cases (48.39%) displayed brain lesions, primarily located in the subcortical, periventricular lateral ventricles, pons, and cerebellum, presenting a patchy T2 hyperintense signal, with or without enhancement. Furthermore, 27 cases (43.55%) had optic neuropathy, involving optic crossings, optic tracts, and lateral geniculate nuclei, and the lesions showed segmental, diffuse, or patchy T2 high signals, mostly with enhancement. All patients underwent at least one positive serum anti-MOG antibody test. Among the 54 patients who underwent CSF examination, 40 (74.07%) had increased CSF leukocyte counts, 27 (50.00%) had increased CSF protein levels, and 5 (9.26%) had elevated CSF pressures. Regarding treatment, 57 (86.36%) received corticosteroids, 15 (22.73%) received immunoglobulin, and 9 (13.64%) received both. Most patients (87.88%) had a favorable prognosis after receiving corticosteroids and immunoglobulins (detailed information is given in Table 3).

**Table 3:** Clinical characteristics of infections combined with MOGAD MOGAD: Myelinating oligodendrocyte glycoprotein antibody-associated disease

Characteristics	Available values	Characteristics	Available values
Sex	n=66	Eye pain	15 (22.73%)
Male	39 (59.09%)	Ataxia	10 (15.15%)
Female	27 (40.91%)	Limb weakness	8 (12.12%)
Source of infection	n=66	Hallucinations	3 (4.55%)
COVID-19	33 (50.00%)	Dysarthria	2 (3.03%)
COVID-19 vaccination	5 (7.57%)	Dysphagia	1 (1.52%)
Mycoplasma pneumoniae	4 (6.06%)	MRI examination	n=62
HIV, varicella-zoster virus	3 (4.55%)	Spinal cord	38 (61.29%)
Cytomegalovirus, influenza A, and Epstein-Barr	2 (3.03%)	Brain	30 (48.39%)
Others	1 (1.52%)	Optic neuropathy	27 (43.55%)
Clinical manifestations	n=66	Antibody test	n=66
Optic neuritis	19 (28.79%)	Serum anti-MOG antibody	66 (100%)
Acute disseminated encephalomyelitis	15 (22.73%)	CSF examination	n=54
Myelitis	15 (22.73%)	Leukocytosis	40 (74.07%)
Neuromyelitis optica	8 (12.12%)	Proteinosis	27 (50.00%)
Encephalitis	7 (10.61%)	Intracranial hypertension	5 (9.26%)
Meningoencephalitis	2 (3.03%)	Treatments	n=66
Clinical symptoms	n=66	Corticosteroids	57 (86.36%)
Fever	33 (50.00%)	Immunoglobulin	15 (22.73%)
Visual disturbances	29 (43.94%)	Corticosteroids + Immunoglobulin	9 (13.64%)
Urinary retention	27 (40.91%)	Outcomes	n=66
Sensory disturbances	24 (36.36%)	Complete response	58 (87.88%)
Paraplegia	21 (31.82%)	Disability	5 (7.58)
Headache	17 (25.76%)	Death	3 (4.55%)

Previous research has demonstrated that various pathogens (such as viruses, bacteria, fungi, Mycoplasmas, Chlamydia, and parasites) can directly harm organs and tissues, prompting a robust immune response to eliminate foreign intruders swiftly. Simultaneously, pathogen infections can induce multiple inflammatory reactions, leading to cell attraction, differentiation, and proliferation within the innate and adaptive immune systems. Additionally, pathogen infections can trigger autoimmune responses primarily through pathways such as molecular mimicry, bystander activation, superantigens, and epitope spreading [12,13].

Molecular mimicry is one of the most popular theories of infection-induced autoimmunity. It occurs when a pathogen's infectious peptide has a similar amino acid sequence to that of a human peptide, inducing the production of cross-reactive antibodies and effector immune cells, which can destroy human cells through autoimmune mechanisms [14]. In cases of infection combined with MOGAD, pathogenic bacteria such as COVID-19, mycoplasmas, influenza A viruses, varicella-zoster virus, and chlamydia can induce the body to produce anti-MOG antibodies through molecular mimicry mechanisms [14-17]. The pathogenesis of Zika virus infection combined with MOGAD may involve inflammation-induced disruption of the blood-brain barrier, leading to the leakage of MOG antigens into the peripheral circulation. It may also stimulate peripheral adaptive immune responses that target MOG, resulting in the production of specific autoimmunity against MOG proteins [18].

Additionally, infection-associated cytokine and chemokine markers are important components of MOGAD. Under inflammatory conditions in the peripheral blood, MOG-specific T cells, especially CD4+ T cells, may be activated through bypass activation systems or molecular mimicry mechanisms; activated T cells further lead to opening of the blood-brain barrier, which allows autoantibodies and immune cells to enter the CNS, causing demyelination [2]. Postinfectious cytotoxicity can also lead to demyelination of oligodendrocytes through a mechanism that alters the membrane potential and inhibits potassium channels [19]. 

The treatment of infections combined with MOGAD involves initiating immunotherapy while treating specific pathogens. In the acute phase, high-dose corticosteroids are the first choice, followed by gradual reduction and continued small doses. Additionally, intravenous immunoglobulin or plasma exchange may be used. However, a multicenter study revealed that patients who received immunoglobulin therapy had a lower relapse rate [20]. Maintenance during remission often includes oral steroids, azathioprine, mycophenolate, and B-cell-targeted biologics (e.g., rituximab and ocrelizumab) [7]. The decision to administer immunotherapy depends on various factors such as the patient's response to the first episode, the severity of the first episode of symptoms, the short-term risk of disability, the long-term risk of immunotherapy, and the patient's age [1]. Regular monitoring and follow-up are important for assessing the effectiveness and safety of treatment.

Furthermore, there is increasing recognition of the importance of creating personalized treatment plans for patients with MOGAD. Adapting treatment approaches according to the individual patient's circumstances, such as age, sex, any other health issues, and lifestyle, can increase the effectiveness of treatment and reduce potential side effects. It is crucial not to neglect the mental well-being of patients in clinical practice. Patients often experience emotional stress and anxiety due to the impact of MOGAD-related dysfunction on their daily lives. Thus, providing psychological counseling and support can assist them in coping with the disease and enhancing their overall quality of life.

## Conclusions

In conclusion, various types of pathogenic infections such as viral, bacterial, mycoplasma, and chlamydia infections, can be associated with MOGAD. Its primary clinical symptoms include optic neuritis, myelitis, and acute disseminated encephalomyelitis. Therefore, timely antibody testing is crucial when patients present relevant symptoms. To increase diagnostic accuracy, it is recommended to prioritize serum antibody testing. Additionally, rational and comprehensive electrophysiological and imaging tests are essential to avoid the potential negative effects of early treatment on test results. Upon confirmation of MOGAD diagnosis, immediate initiation of immunotherapy is necessary to improve the patient's overall prognosis.
